# DNA methylation signatures of chronic alcohol dependence in purified CD3^+^ T-cells of patients undergoing alcohol treatment

**DOI:** 10.1038/s41598-017-06847-z

**Published:** 2017-07-26

**Authors:** Christof Brückmann, Sumaiya A. Islam, Julia L. MacIsaac, Alexander M. Morin, Kathrin N. Karle, Adriana Di Santo, Richard Wüst, Immanuel Lang, Anil Batra, Michael S. Kobor, Vanessa Nieratschker

**Affiliations:** 1 Department of Psychiatry and Psychotherapy, University Hospital of Tuebingen, Tuebingen, Germany; 2 Department of Medical Genetics, University of British Columbia, Vancouver, BC Canada; 3 Centre for Molecular Medicine and Therapeutics, BC Children’s Hospital, Vancouver, BC Canada; 4 Department of Neurodegenerative Disease, Hertie-Institute for Clinical Brain Research, Tuebingen, Germany; 5 Human Early Learning Partnership, University of British Columbia, Vancouver, British Columbia Canada; 6 Canadian Institute for Advanced Research, Toronto, Ontario Canada

## Abstract

Several studies have shown an association of alcohol dependence with DNA methylation (DNAm), suggesting that environmentally-induced changes on epigenomic variation may play an important role in alcohol dependence. In the present study, we analysed genome-wide DNAm profiles of purified CD3^+^ T-cells from pre- and post-treatment alcohol dependent patients, as well as closely matched healthy controls. We identified 59 differentially methylated CpG sites comparing patients prior to treatment with healthy controls and were able to confirm 8 of those sites in additional analyses for differentially methylated regions. Comparing patients before and after a 3-week alcohol treatment program we revealed another unique set of 48 differentially methylated CpG sites. Additionally, we found that the mean global DNAm was significantly lower in patients prior to treatment compared to controls, but reverted back to levels similar to controls after treatment. We validated top-ranked hits derived from the epigenome-wide analysis by pyrosequencing and further replicated two of them in an independent cohort and confirmed differential DNAm of *HECW2* and *SRPK3* in whole blood. This study is the first to show widespread DNAm variation in a disease-relevant blood cell type and implicates *HECW2* and *SRPK3* DNAm as promising blood-based candidates to follow up in future studies.

## Introduction

Alcohol dependence (AD) is a severe disorder that has long-lasting detrimental consequences, resulting in considerable health, economic and societal burden. According to the World Health Organization, alcohol related diseases account for approximately 3.3 million deaths per year (WHO, 2014). Although this number is alarmingly high, studies indicate that problematic drinking behaviour still is underestimated^1^. To date, treatment options are limited and the effectiveness of existing alcohol treatment programs is often less than optimal or difficult to assess, warranting a need for improvement.

The pathogenesis of AD is complex and includes genetic as well as non-genetic factors. Evidence is emerging that the interaction between underlying genetic factors and environmental stimuli (gene x environment, GxE) in particular plays a major role in addiction-related disease states^2–4^. Such findings have prompted considerable inquiry into the biological basis of GxE influences, with epigenetic regulation providing one of the most compelling candidate mechanisms for the mediation of GxE effects^5, 6^.

One of the most frequently studied epigenetic mechanisms is DNA methylation (DNAm), which involves the covalent addition of a methyl group to the 5′ position of a cytosine, primarily in the context of a cytosine-phosphate-guanine (CpG) dinucleotide. CpG dinucleotides are especially prevalent in CpG islands, genomic regions of approximately 1000 base pairs (bp) with a CG content greater than 50%^7^. CpG islands are associated with 50–70% of human gene promoters and increased DNAm in these regions is generally correlated with a decreased transcription of the respective gene^8, 9^. Furthermore, methylated regions adjacent to CpG islands, called CpG island shores (up to 2 kb in either direction) or shelves (from 2 to 4 kb in either direction), may contribute to and potentiate epigenetic effects on gene expression^10–12^. In recent years, there has been increasing appreciation for the complexity of the relationship between DNAm and gene expression regulation, which tends to be highly dependent on genomic context^9, 13^. DNAm profiles of genetic regions can vary substantially between different cell types^14^. It has been shown that after tissue origin, cellular heterogeneity within a tissue is a major driver of DNAm variance, highlighting the need to account for cellular composition in DNAm analyses^15, 16^.

Several biological factors including age^17^, sex^18^ and ethnicity^19^ also have a profound impact on DNAm patterns. In addition, a number of lifestyle-based environmental exposures, including smoking^20–23^ and alcohol consumption^24–36^, are associated with variation in DNAm. In particular, DNAm alterations in AD patients have been documented in a number of epigenetic studies in human populations. For example, candidate gene analyses reported differential DNAm of the dopamine^30^ and serotonin transporters^32^, the nerve growth factor *NGF* ^27^, leptin^28^ and most recently *GDAP1* ^25^ in AD patients compared to healthy controls. In the context of epigenome-wide association studies (EWAS), previous studies found widespread AD-associated DNAm differences at single sites, differentially methylated regions (DMRs)^26, 33–35^ and in “bulk” DNAm, representing mean global total levels of DNAm^29, 36^. One study assessed DNAm alterations in peripheral blood mononuclear cells (PBMCs) of AD patients participating in a short-term alcohol treatment program compared to healthy controls, and reported differential methylation at 56 CpG sites in patients prior to treatment compared to controls. Although no statistically significant DNAm differences were observed in patients before and after the alcohol treatment program, 49 of the 56 differential sites reverted back in patients post-treatment to levels similar to controls^31^. Together, these previous studies identified a multitude of AD-associated differentially methylated sites, however, they did not account for cell type heterogeneity in their analyses, thereby potentially resulting in associations that are confounded by inter- and intra-individual differences in cellular composition. Most recently, a study involving 13,317 participants from 13 distinct cohorts analysed DNAm profiles in monocytes and whole blood. This analysis, which was adjusted for cell composition, revealed hundreds of AD-associated differentially methylated CpG sites^29^.

Although all these previous studies support a potential link between DNAm variation and AD, a number of questions have yet to be explored: I) Are there signatures of AD in a disease-relevant blood cell type? II) Does treatment result in reversion of differential DNAm back to the levels found in controls? III) Importantly, can such AD-associated differential DNAm be replicated in independent cohorts, signifying the robustness of the identified genome-wide hits, and IV) Can the differential DNAm from a purified blood cell type also be detected in whole blood samples, indicating the potential relevance of these associations in other blood cell types?

To address these questions, we assessed genome-wide DNAm profiles of purified CD3^+^ T-cells of a well-characterized cohort of long-term chronic AD patients participating in a clinical 3-week alcohol treatment program, along with the profiles of healthy controls closely matched for sex, age, ethnicity and smoking behaviour. We restricted our analyses to T-cells due to the known effects of chronic alcohol abuse in modulating the number, activity and relative subtype abundance levels of these immune cells^37^. For example, short-term binge drinkers as well as chronic AD patients exhibit a reduced number of peripheral T-cells^38^. In addition, a shift from CD4^+^ and CD8^+^ naïve T-cells towards memory T-cells is observed in AD patients^39^. Furthermore, alcohol consumption influences T-cell activation, leading to elevated numbers of activated CD8^+^ T-cells, which may contribute to chronic inflammation^37, 40^. For these reasons, heightened susceptibility to infections, including tuberculosis, pneumonia and HIV is observed in those patients^37, 41^. T-cells have also been used previously in similar epigenetic studies due to their regulatory function in neuroimmune mechanisms^42, 43^. Furthermore, by comparing the patients before and after 3 weeks of participating in a clinical alcohol treatment program, we sought to identify differentially methylated sites that may play a potential role in alcohol withdrawal and early recovery. In order to test whether our findings were robust, we validated four of our top-ranked hits by pyrosequencing, replicated the top-ranking hits in an independent second cohort of AD patients and matched controls and additionally confirmed the top-ranking hits in whole blood DNA of our cohort samples.

## Results

### Study cohorts and DNA methylation array normalization

To identify AD-associated DNAm variation, we utilized a discovery and replication cohort of AD patients and healthy controls, who were closely matched for age, sex and smoking behaviour. Demographic and AD-relevant characteristics as well as AUDIT and GSI scores of both cohorts are provided in Table 1a and b. To measure the effectiveness of the 3-week alcohol treatment program, we compared both GSI and OCDS scores in the discovery cohort at the beginning and after treatment. We found that both values decreased significantly, indicating a reduced alcohol craving and a better overall psychological well-being post-treatment (Table 1c).

**Table 1:** Description of a) the discovery study cohort, b) the replication study cohort and c) results after 3-week alcohol treatment program in the discovery cohort.

	a) Discovery study cohort			b) Replication study cohort		
	Controls (N = 23)	Patients (N = 24)	*P*-value	Controls (N = 12)	Patients (N = 13)	*P*-value
age	46.9 ± 10.3	47.5 ± 10.1	0.8	45.3 ± 16.2	50.9 ± 9.1	0.4
active smokers	18 (78%)	19 (79%)	0.9	8 (67%)	9 (69%)	0.9
cigarettes per day	13.8 ± 12.6	15.2 ± 10.7	0.7	8.9 ± 8.0	10.5 ± 9.4	0.7
Years of alcohol dependence		10.6 ± 9.4			14.6 ± 11.7	
Days since last drink before hospital admission		1.2 ± 0.6			0.3 ± 0.4	
Standard drinks consumed each day in the week before hospital admission		13.7 ± 8.3			19 ± 11.4	
AUDIT	5.9 ± 3.8	24 ± 6.5	4E-15	2.8 ± 2.3	28.0 ± 4.9	3E-14
GSI	0.15 ± 0.14	0.72 ± 0.45	6E-07	0.10 ± 0.09	0.11 ± 0.10	0.9
	**c) Results after 3-week alcohol treatment in the discovery cohort**					
	**Patients (T1)**		**Patients (T2)**		***P*** **-value (paired testing)**	
GSI	0.72 ± 0.45		0.41 ± 0.52		0.036	
OCDS	19.3 ± 6.6		12.0 ± 4.9		3E-05	

Errors are given as standard deviation. Abbreviations: AUDIT, alcohol use disorder identification test; GSI, global severity index; OCDS, obsessive compulsive drinking scale.

In order to assess the association of AD with genome-wide DNAm in our discovery cohort, we measured site-specific DNAm at over 450,000 CpGs using the Illumina 450 K array. To test for potential cellular heterogeneity in the bead-purified CD3^+^ T-cell samples, we used the Houseman blood deconvolution algorithm to estimate cell-type proportions, observing up to 32% of contaminating non-T-cell DNA in a fraction of our samples, although these proportions were not correlated with group status (Supplementary Figure S1). Regression-based adjustment of 450 K data resulted in the removal of these cell type associations as assessed by PCA (Supplementary Figure S2). The adjusted dataset thereby represented DNAm profiles from T-cells whose inter-individual cell type differences had been normalized to the best of our abilities for subsequent analyses.

### Identification of AD-associated differential DNAm

**Figure 1:**
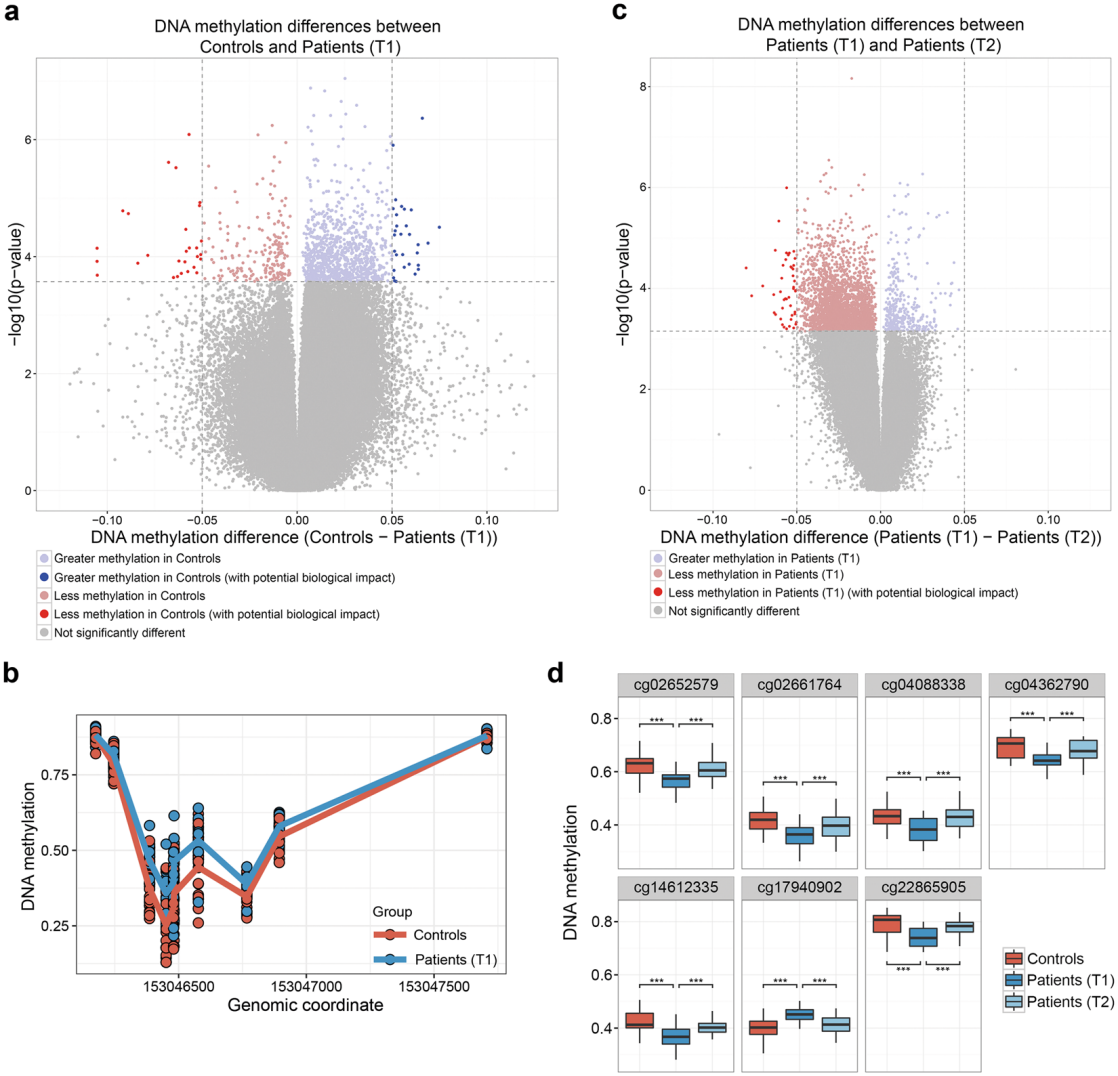
Differential sites and regions identified in the 450 K array analyses. (**a**) Volcano plot depicting differences in DNAm levels between controls and patient (T1) for each probe in the corrected 450 K dataset (indicated on X axis) against FDR (indicated on Y axis, on –log10 scale). Dashed horizontal line denotes FDR threshold of 0.1 while dashed vertical lines denote DNAm difference thresholds of −0.05 and 0.05, respectively. (**b**) Differential DNAm detected by DMRcate in the promoter region of the *SRPK3* gene (chrX:153, 046, 386–153, 046, 482). (**c**) Volcano plot depicting differences in DNAm levels between patients (T1) and patients (T2) as described in panel (a). (**d**) DNAm levels of seven sites which show reversion of DNAm post-treatment. ***Indicate an FDR < 0.001.

Based on site-specific analyses of the T-cell DNAm array profiles, we identified 59 differentially methylated CpG sites between patients (T1) and controls with DNAm differences (Δ-beta) of at least 5% to increase the likelihood of biological relevance (FDR < 0.1). Of these 59 hits, 28 sites showed higher methylation, while 31 sites had lower methylation in patients compared to controls. Differences in DNAm ranged from 5 to 14% (Fig. 1a). The top 10 hits, ranked by Benjamini-Hochberg (BH)-adjusted *P*-value significance, are listed in Table 2a. A complete list of all 59 significant hits (FDR < 0.1) is provided in Supplementary Table S1. The top-ranked hit (cg18752527) exhibited a DNAm difference of 6.6% and was located within the intragenic region of the *HECW2* gene.

**Table 2:** Top 10 differentially methylated sites a) between controls and patients (T1) and b) between patients (T1) and patients (T2).

Probe ID	Gene	Region	Average beta Controls	Average beta Patients (T1)	Δ-beta	*P-*Value	BH-adjusted *P*-value
**a) Differentially methylated sites between Controls and Patients (T1)**							
cg18752527*	*HECW2*	intragenic	0.342	0.276	0.066	4.30E-07	0.0213
cg08109624		intergenic	0.760	0.817	−0.057	8.15E-07	0.0234
cg10168086		intergenic	0.535	0.484	0.051	1.24E-06	0.0256
cg07280807*		intergenic	0.755	0.822	−0.068	2.44E-06	0.0366
cg12173150		intergenic	0.321	0.385	−0.064	3.02E-06	0.0370
cg01059398	*TNFSF10*	intragenic	0.261	0.209	0.052	1.07E-05	0.0627
cg17940902	*HLA-DMA*	promoter	0.399	0.450	−0.051	1.19E-05	0.0640
cg22778903	*MX2*	intragenic	0.304	0.355	−0.051	1.34E-05	0.0666
cg14612335	*SKIL*	promoter	0.423	0.368	0.055	1.38E-05	0.0666
cg11580026		intergenic	0.600	0.549	0.051	1.51E-05	0.0691
**Probe ID**	**Gene**	**Region**	**Average beta Patients (T1)**	**Average beta Patients (T2)**	**Δ-beta**	***P*** **-Value**	**BH-adjusted** ***P*** **-value**
**b**) **Differentially methylated sites between Patients** (**T1**) **and Patients** (**T2**)							
cg15500907	*LAMA4*	intragenic	0.485	0.542	−0.056	1.01E-06	0.0323
cg05266321	*CCR2*	intragenic	0.545	0.606	−0.061	4.63E-06	0.0487
cg13279700	*C6orf10*	intragenic	0.481	0.544	−0.063	1.76E-05	0.0561
cg14054990	*KRTAP19-5*	promoter	0.431	0.482	−0.052	1.84E-05	0.0565
cg21049302		intergenic	0.466	0.522	−0.056	1.98E-05	0.0565
cg17022548	*NRG2*	intragenic	0.204	0.258	−0.054	1.99E-05	0.0565
cg22472360	*TRIO*	intragenic	0.514	0.569	−0.055	2.09E-05	0.0569
cg07920414	*RIMS3*	intragenic	0.438	0.493	−0.055	2.18E-05	0.0572
cg04088338		intergenic	0.378	0.429	−0.051	2.54E-05	0.0590
cg12240358	*HOMER2*	intragenic	0.462	0.519	−0.057	2.68E-05	0.0590

Probe IDs marked with an asterisk were validated by pyrosequencing. Abbreviations: Average beta, mean methylation values (%); Benjamini-Hochberg (BH) adjusted *P*-value.

In addition to single CpG sites, we identified 29 significant DMRs (FDR < 0.01, Δ-beta > 5%) using DMRCate. These DMRs contained 153 CpG sites, of which 8 were also identified as differentially methylated in the site-specific analysis between controls and patients (T1) (Supplementary Table S2). Interestingly, 4 of these overlapping 8 hits were in the *SRPK3* gene region (Fig. 1b).

### Treatment-related alterations in T-cell DNAm profiles

To identify differentially methylated sites potentially playing an important role in alcohol withdrawal and early recovery in AD patients, we compared genome-wide T-cell DNAm profiles of patients before (T1) and after 3 weeks (T2) of participation in an alcohol treatment program. Using paired testing in our site-specific analyses, we identified 48 differentially methylated sites between patients (T1) and patients (T2), all of which showed increased methylation at T2 ranging from 5 to 12% difference (FDR < 0.1, Δ-beta > 5%) (Fig. 1c, Supplementary Table S3). The top 10 hits are listed in Table 2b. Utilizing the same threshold as before, we did not observe any DMRs in patients before and after treatment.

### Post-treatment reversion of differentially methylated sites

To examine whether AD-associated DNAm is influenced by a 3-week alcohol treatment program, we assessed DNAm levels in patients post-treatment at the 59 sites identified in the analysis comparing controls and patients (T1). After the treatment (T2), the DNAm levels of 7 out of 59 sites reverted back to a level where they no longer significantly differed from controls (Fig. 1d). Based on paired testing, we determined that these 7 sites were indeed differentially methylated between patients (T1) and patients (T2). Moreover, 32 CpG sites showed a trend to revert back, though not significant at an FDR < 0.1. The DNAm levels of the remaining 20 sites did not change from T1 to T2.

### Assessment of mean global DNAm differences between groups

**Figure 2:**
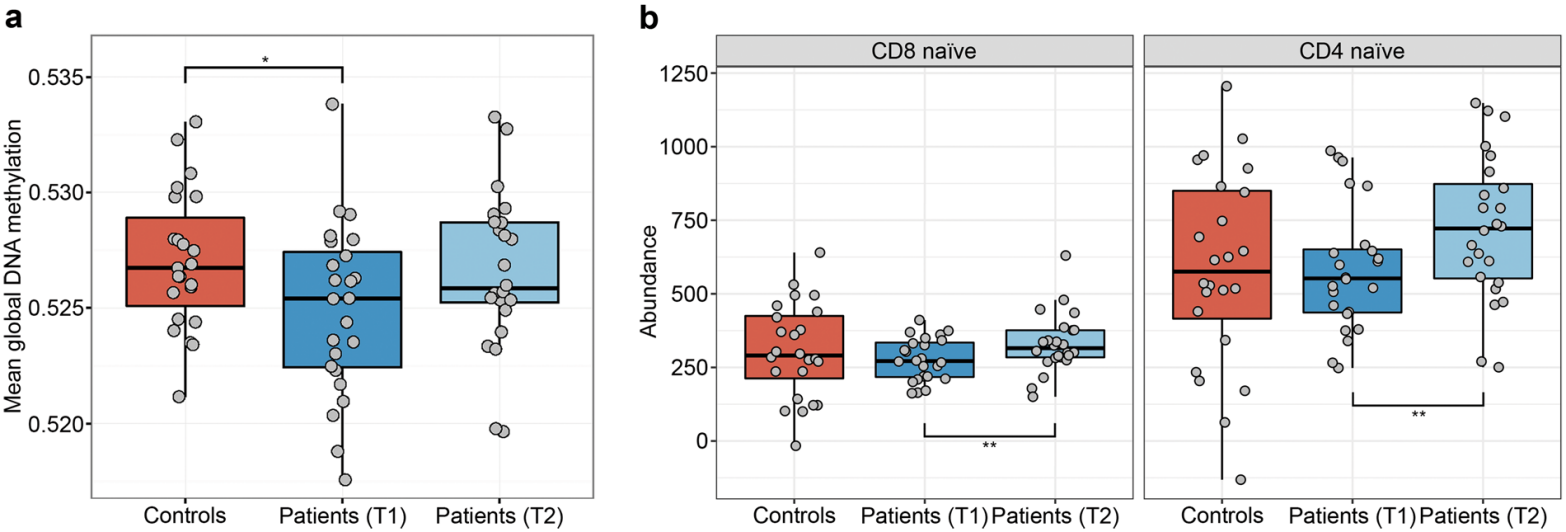
Mean global DNAm differences and naïve T-cell subtype differences between groups. (**a**) Patients (T1) showed significantly decreased mean global DNAm levels compared to controls (P = 0.048, Mann-Whitney U test). Differences between controls vs. patients (T2) and patients (T1) vs. patients (T2) were not significant. (**b**) Abundance levels of naïve CD8^+^ and CD4^+^ T-cells were predicted using an advanced blood DNA methylation age prediction tool. Both naïve T-cell subtypes significantly increased post-treatment in patients (**Indicates an FDR < 0.01, Wilcoxon signed-rank test) but were not significantly different between controls and patients at either time point.

Given the unidirectional change in our site-specific analysis of patients before and after treatment, particularly at AD-associated sites which showed post-treatment reversion, we next examined if this trend was related to AD-associated differences in mean global DNAm. Here we defined mean global DNAm as the calculated average of DNAm values across all sites in each sample. We found that although the result was only nominally statistically significant, prior to the alcohol treatment (T1), mean global DNAm was lower in patients compared to controls (*P* = 0.048, Mann-Whitney U test). However, at the end of treatment (T2), global DNAm of the patients approximated the levels seen in controls and no longer differed significantly from controls (Fig. 2a). This finding was consistent with the unidirectional differences, in that all significant sites between patients before and after treatment showed increased methylation at T2 in the site-specific analysis, and supported the observed post-treatment reversion of AD-associated sites. Of note, these differences in mean global DNAm are unlikely to be driven by batch effects or other sources of technical variation due to the fact that all samples were run in a randomized manner on the same set of arrays.

### Differences in naïve T-cell subtype abundances between groups

To evaluate if there were differences in underlying T-cell subtypes between the groups, we estimated abundance measures of additional blood cell subsets using an advanced blood analysis option for an epigenetic clock prediction tool^44^ on our T-cell 450 K profiles. We observed that the predicted abundance levels of both CD4^+^ and CD8^+^ naïve T-cell subsets significantly increased post-treatment in AD patients (FDR < 0.01, Wilcoxon signed rank test) (Fig. 2b). However, the abundance of these naïve T-cell subtypes was not statistically significantly different between controls and patients at either time point.

### Validation of AD-associated differential DNA methylation by pyrosequencing

**Figure 3:**
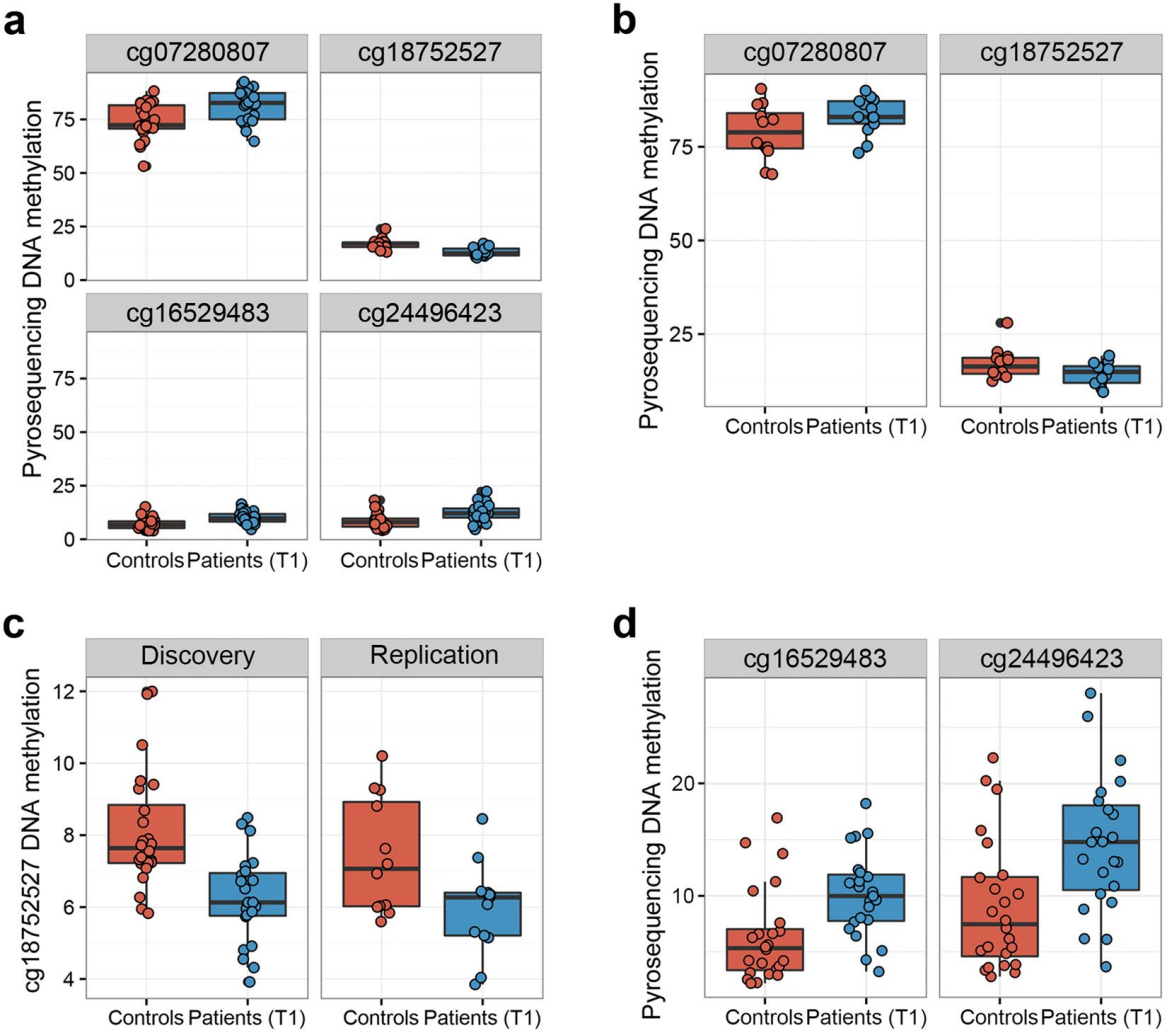
Validation and replication of top-ranking hits by pyrosequencing. (**a**) Boxplots showing differences in DNAm levels of discovery cohort T-cell samples as measured by pyrosequencing (FDR < 0.01, Student’s t-test). (**b**) Two top-ranked hits (cg07280807 and cg18752527) were verified as being differentially methylated in T-cell samples of the replication cohort (FDR < 0.05, one-sided t-test). (**c**) Verification of differential methylation of cg18752527 (*HECW2*) in the discovery (left) and the replication cohort (right) in DNA derived from whole blood (FDR < 0.05, two-sided t-test). (**d**) Verification of cg16529483 and cg24496423 (*SRPK3*) differential methylation in the discovery cohort in DNA derived from whole blood (FDR < 0.01, two-sided t-test).

To verify the results from the 450 K dataset, we selected two top-ranking differentially methylated sites between controls and patients (T1) (cg18752527 in the *HECW2* gene and cg07280807 in an intergenic region) for validation using pyrosequencing as an independent readout of DNAm measures. We additionally validated two promoter CpGs of *SRPK3* (cg16529483 and cg24496423) since differential methylation in the *SRPK3* gene region was found to be a robust finding in our DMRcate analyses. We were able to confirm significant differences between controls and patients (T1) at all 4 sites, as shown in Fig. 3a (Student’s t-test, FDR < 0.01). Although Bland-Altman plots showed a general bias for lower methylation levels measured by pyrosequencing (Supplementary Figure S3), the correlation in measurements between the two methodologies was highly concordant for all 4 sites (Spearman’s correlation r_s_ > 0.7, FDR < 0.001) (Supplementary Figure S3).

### Replication of AD-associated differential DNA methylation in an independent cohort

To further test the robustness of our EWAS findings, we analysed the previously mentioned 4 sites in T-cells of an independent replication cohort by pyrosequencing. The two top-ranking hits, cg07280807 in an intergenic region and cg18752527 in *HECW2*, were differentially methylated in the replication cohort (FDR < 0.05, one-sided t-test) (Fig. 3b). However, the two sites within the *SRPK3* promoter region (cg16529483 and cg24496423) did not replicate in this cohort, likely due to insufficient power with the low sample size in this cohort, but showed a trend in the same direction as in the discovery cohort.

### Analysis of differential DNA methylation in whole blood

To identify sites that are not only differentially methylated in T-cells, but also in whole blood DNA, we sought to reproduce our most robust EWAS findings from T-cells in whole blood DNA samples of both our discovery and replication cohorts. Therefore, we analysed DNAm of the 4 previously mentioned sites in whole blood samples by pyrosequencing. We observed differential methylation of cg18752527 in the intragenic region of *HECW2* between controls and patients (T1) in both cohorts (FDR < 0.05, Student’s t-test) (Fig. 3c). Furthermore, similar to the findings from T-cells, the two sites within the *SRPK3* promoter region (cg16529483 and cg24496423) were differentially methylated in whole blood samples of the discovery cohort (Fig. 3d), but not of the replication cohort. We found that differential DNAm of cg07280807 did not replicate in whole blood of either cohort. Using a previous 450 K dataset of purified blood cell types^45^, we confirmed that the DNAm status of cg18752527 in *HECW2* was highly associated with T-cells, along with NK cells, suggesting that the DNAm differences we measured in whole blood were driven, in part, by T-cells (*P* = 7.6E-15, ANOVA) (Supplementary Figure S4). The DNAm statuses of the two sites in the *SRPK3* promoter were not associated with any specific cell type (Supplementary Figure S4).

## Discussion

By analysing genome-wide DNAm profiles of purified CD3^+^ T-cells using the Illumina 450 K array, we found 59 CpG sites to be differentially methylated in a group of 24 alcohol dependent patients compared to 23 closely matched healthy controls. These site-specific hits showed considerable overlap to detected DMRs, suggesting that the results were not contingent on the analytical approach used. Furthermore, we discovered 48 sites that were differentially methylated between AD patients at the time of hospital admission (T1) and after 3 weeks (T2) of participation in an alcohol treatment program and showed a reversion of some of the AD-associated sites post-treatment. In addition, we were able to validate four of the top-ranking AD-associated hits by pyrosequencing, and replicate two of them in an independent cohort. Finally, we found the top-ranked hits in *HECW2* (cg18752527) and *SRPK3* (cg16529483 and cg24496423) to be differentially methylated in whole blood, signifying the potential relevance of these associations in other blood cell types. To our knowledge, this is the first study to assess and replicate alcohol-associated differential DNAm in purified T-cells and to assess DNAm variation that may be related to early recovery from AD in closely matched human population cohorts.

EWAS pose an excellent hypothesis-free opportunity to identify as yet undiscovered disease-associated genes. Our EWAS findings of AD-associated differential DNAm revealed both site-specific and regional differences between patients before treatment and matched controls in a clinically relevant cell type. The observed bi-directional patterns of changes are consistent with previous evidence of AD-associated differential DNAm^26, 29, 31, 33, 35^. However, our findings derived from T-cells did not overlap with previously reported associations of AD with DNAm^26, 29, 31, 33, 35^. This can at least in part be explained by the use of heterogeneous biological material (i.e. whole blood, PBMCs), differences in the cohorts used or in the strategies applied to match patients and controls as well as by varying methodologies for DNAm measurement, with reduced or discordant coverage of CpG sites in previous studies^26, 31, 33, 35^ compared to the present study. However, our top-ranking hits in *HECW2* and *SRPK3* might contribute to reveal mechanisms that may play a role in AD. HECW2 is a HECT-type E3 ubiquitin ligase involved in the cellular stress response^46, 47^. This finding is in line with previous evidence for the role of epigenetic regulation of cellular stress response genes in AD, such as *GDAP1*, which was identified in a previous EWAS^31^ and subsequently replicated in whole blood samples derived from an independent cohort^25^. However, *GDAP1* did not come up in this present analysis using DNA isolated from purified T-cells. Presumably, the previously described differential methylation of *GDAP1* in whole blood is driven by another cell type other than T-cells. *SRPK3* encodes a serine/arginine protein kinase and is essential for the development of the skeletal muscle^48^. It was shown that the drosophila homolog *SRPK79D* plays an important role in the function of synapses^49^. Although an association between *SRPK3* and the nervous system in humans has not been described so far, the high homology between SRPK79D and SRPK3 (65%) makes an as yet uncharacterized role in the nervous system possible.

In addition to the assessment of AD-associated differential DNAm in T-cells prior to alcohol treatment, we also examined treatment-related site-specific alterations in DNAm by comparing DNAm profiles in T-cells of patients before (T1) and after a 3-week alcohol treatment (T2). Our findings include numerous sites in which DNAm in patients (T2) reverts back to levels comparable to those observed in controls. More specifically, we showed post-treatment DNAm reversion (at 7 sites) or partial reversion (at 32 sites) back to control levels. These findings confirm the results of a previous pilot study, which also showed reversion of DNAm after a short term alcohol treatment program^31^. Other epigenetic studies in human populations investigating the effect of short-term treatments, including exercise or dietary interventions, on DNAm of relevant tissues have identified similar numbers of site-specific DNAm changes with a comparable magnitude of effect sizes to our findings^50, 51^.

Based on our assessment of mean global DNAm, measured as averaged methylation across all interrogated CpGs, we found that global DNAm levels were significantly lower in patients prior to the alcohol treatment compared to controls. Following alcohol treatment, the mean global DNAm of patients no longer differed significantly from controls. These results are in accordance with the unidirectionality of our treatment-related hits, with all significant sites exhibiting increased DNAm after treatment, and with our site-specific findings that numerous AD-associated CpGs exhibited post-treatment reversion to levels comparable to controls. The reduction in mean global DNAm observed in AD patients is supported by previous studies, which also demonstrated decreased methylation^29, 36^. It has been hypothesized that such alcohol-associated decreases in global DNAm are attributed to the lack of methionine adenosyl transferase regulation in AD patients^14, 52^. However, in contrast, earlier studies have postulated that due to the higher homocysteine levels in AD patients, global DNAm patterns should be elevated^53^, although such associations have not been confirmed^54^. The lack of consensus in regard to alterations in alcohol-related global DNAm measures highlights the need for further investigation into the biological mechanisms underlying global DNAm patterns in AD patients.

Using bioinformatic predictions from our T-cell DNAm profiles, we observed a significant increase in naive CD4^+^ and CD8^+^ T-cell subsets post-treatment, which is consistent with evidence of decreased frequencies of these naïve T-cell subtypes due to chronic AD^37, 39^ and a resultant restoration of peripheral T-cell numbers following short-term alcohol abstinence^38^. These findings, along with known effects of alcohol dependence on T-cell homeostasis, proliferation and activation^39, 55^, highlight the importance of understanding alcohol-related effects on T-cell-specific biology, particularly in the context of AD pathophysiology and treatment, of which our study serves as the first to profile such AD-associated changes on the T-cell epigenome.

In order to verify that our results are robust and largely reflective of potential biological variation as opposed to technical variation, we took a number of precautions in our analyses, including I) constraining our hits to sites with DNAm differences greater than 5% between groups in order to increase the likelihood of biological relevance, II) confirming 450 K measures by pyrosequencing and III) validating top-ranked hits by pyrosequencing in an independent replication cohort. Although we observed a general bias between the two methodologies, in which the pyrosequencing measures were lower than 450 K values, there was high concordance of measures between the two methods and we were still able to detect significant differences in DNAm between groups, signifying the strength of our results. Moreover, we were able to confirm three top-ranking hits from purified T-cells in whole blood, further strengthening the robustness of our findings and highlighting their potential importance in AD.

It is important to note that our study had a few inherent limitations. Firstly, using bioinformatic cell type predictions, we detected notable levels (up to 32%) of cellular contamination in our bead-purified T-cell samples. This is consistent with previous work which confirmed the presence of cellular heterogeneity in samples even after purification using cell surface markers^56^. We removed cell heterogeneity using a regression-based method, thereby ensuring inter-individual differences in cell composition were normalized in our dataset prior to DNAm analyses. Secondly, our analyses were limited by a rather small sample size. To work around this limitation, we utilized a relaxed FDR threshold in the differential methylation modelling to capture more potentially biologically relevant sites and focused on validating and replicating our top-ranked hits to ensure these results were robust. Although we were able to validate the hits within the *SRPK3* promoter by pyrosequencing in T-cell and whole blood samples of the discovery cohort, we could not replicate the differential DNAm of *SRPK3* in our second cohort, unlike our findings for *HECW2*. This probably results from insufficient statistical power due to the low sample size of the replication cohort. We acknowledge that the small samples size analysed in our study could also hinder successful validation of our results in future studies. The phenomenon of non-replication could also be observed in previous transcriptome-wide studies in human populations of AD patients and control individuals, where the overlap between the individual studies was fairly small^57, 58^. However, by technically validating and replicating our results in a second cohort, we made an attempt to reduce the risk of false-positive findings to a minimum. Despite these efforts, our results should be verified in a larger cohort spanning different populations to confirm the associations for *HECW2* and *SRPK3*. So far, neither *HECW2* nor *SRPK3* were among top-ranked hits in transcriptome-wide studies. Therefore, functional data is required to investigate the interplay of DNAm, transcription and functioning of these genes related to AD. Thirdly, we cannot rule out that the DNAm differences between the patients before (T1) and after treatment (T2) may be due to stochastic temporal DNAm variation, although previous work in blood has revealed minimal evidence of temporal variation in the majority of 450 K probes across a 9 month period^59^. In addition, differences in DNAm could also be due to direct influences of acute ethanol intoxication, which has been shown to have an effect on transcriptome regulation^57, 58^. We tried to circumvent this limitation by only including subjects who had their last drink in a narrow time frame of 1.2 ± 0.6 days. Additionally, the 20 CpG sites which did not change from pre- to post-treatment could potentially be differentially methylated due to chronic alcohol exposure and not due to early withdrawal. To clarify this issue, future longitudinal studies are warranted. Finally, we cannot disregard the potential influence of genetic variation on our differentially methylated CpG sites. However, we attempted to reduce genetic heterogeneity in our cohort by using only Caucasian participants.

In conclusion, we report that AD is associated with lower mean global DNAm and with differential DNAm of specific sites in CD3^+^ T-cells. Additionally, we were able to identify changes in DNAm related to alcohol treatment in patients. These changes include the reversion of AD-associated DNAm alterations at certain sites to levels comparable to controls. Validation of our top-ranking associations by pyrosequencing and replication of our top-ranked hits in a second independent cohort strongly supports the robustness of our results. Finally, we show that the differential methylation of *HECW2* and *SRPK3* is not only present in T-cells, but also in whole blood, indicating that *HECW2* and *SRPK3* are likely robust findings which should be followed up in future studies.

## Methods

### Study cohorts

The discovery study cohort was comprised of 24 male AD patients (mean age 47.5 ± 10.1 years) participating in a 3-week in-patient alcohol treatment program at the Clinic for Psychiatry and Psychotherapy in Tuebingen, Germany. AD was diagnosed according to the fourth edition of the Diagnostic and Statistical Manual of Mental Disorders (DSM-IV). Twenty-three population based, sex- and age-matched healthy controls (mean age 46.9 ± 10.3 years) were recruited from Tuebingen and the surrounding area. The replication study cohort was comprised of 13 male AD patients (mean age 50.9 ± 9.1 years) and 12 matched healthy controls (mean age 45.3 ± 16.2 years). In addition, the smoking behaviour (measured as cigarettes per day) of both groups was matched. Subjects with a dependence other than nicotine and patients with any psychiatric disorder necessitating psychotropic medication were excluded from the study. All subjects were of Caucasian origin and gave written informed consent after recovering from alcohol intoxication (patients) or prior to participation in the study (controls), which was approved by the ethics committee of the University of Tuebingen and was conducted in accordance with the Declaration of Helsinki.

After recovery from alcohol intoxication and at the time of study inclusion, respectively (time point 1, T1), patients and controls answered a self-administered phenotypic and demographic questionnaire, the Alcohol Use Disorder Identification Test (AUDIT)^60^, assessing alcohol consumption, and the Symptom Checklist-90-R (SCL-90-R) questionnaire^61^, assessing the global distress level (GSI). Patients also answered the obsessive compulsive drinking scale (OCDS-G) questionnaire, reflecting obsession and compulsivity related to craving and drinking behavior^62^. OCDS-G and SCL-90-R were reassessed after three weeks ( ± 2 days) of participation in the alcohol treatment program (time point 2, T2). Controls with AUDIT scores >15 were excluded, as a higher value is suggestive for problematic alcohol intake.

At T1 and T2, peripheral venous blood was drawn from patients in Ethylenediaminetetraacetic (EDTA) and Mononuclear Cell Preparation tubes (CPT, both BD, Franklin Lakes, NJ, USA). EDTA and CPT blood samples from the controls were drawn at study inclusion. Samples for whole blood DNA extraction were kept at −80 °C until further usage.

### CD3^+^ T-cell purification and DNA isolation

Immediately after blood draw, PBMCs were first separated via centrifugation of the CPT tubes for 20 min at 1650 × g. CD3^+^ T-cells were then purified from PBMCs following the positive isolation protocol using Dynabeads CD3 (Invitrogen, Carlsbad, CA, USA). The cells were subsequently lysed and DNA was prepared using the QIAamp DNA Mini Kit (Qiagen, Hilden, Germany) according to standard protocol.

### Bisulfite conversion and Illumina 450 K DNA methylation arrays

T-cell DNA (750 ng) was bisulfite converted using the Zymo Research EZ DNA Methylation Kit (Zymo Research, Irvine, CA, USA). DNA yield and purity was assessed using a Nanodrop ND-1000 (Thermo Fisher Scientific, Waltham, MA, USA). Samples were subsequently randomized and 160 ng of bisulfite-converted DNA was applied to the Illumina Infinium HumanMethylation450K (450 K) Beadchip array, as per manufacturer’s protocols (Illumina, San Diego, CA, USA).

### DNA methylation array data processing, blood cell deconvolution and differential methylation analyses

Raw data from the 450 K array was subjected to quality control, normalization and batch correction. Subsequently, remaining contamination of the purified T-cells was bioinformatically removed from the dataset. After subsetting the corrected data in 3 groups (controls vs. patients (T1); patients (T1) vs. patients (T2); controls vs. patients (T2)), site-specific differential DNAm was assessed by linear regression modelling while differentially methylated region (DMRs) were identified using the DMRcate package as described earlier^63^. A detailed description of all analyses can be found in the supplementary methods. The 450 K data has been made publicly available on the Gene Expression Omnibus database (GSE98876).

### Pyrosequencing-based validation and replication in T-cells

500 ng T-cell DNA was bisulfite-converted using the Epitect Fast Bisulfite Conversion Kit (Qiagen) as described earlier^25^. For amplification of the region of interest, PCR was conducted using the PyroMark PCR Kit (Qiagen) with the following primers: forward (fwd): 5′-GTTATGGTTGGGTTTTTGGG-3′, reverse (rev): 5′-Bio-CCTATCTCCTCAAACAAAAACTAAAAA-3′, sequencing (seq): 5′-AGTTAGGGATTATAGTGTAGTTG-3′ (cg07280807); fwd: 5′-GTGTTTGTGGGAATGTTTTTTATA-3′, rev: 5′-Bio- CACACTACACTTTCATTTTCTATCAA-3′, seq: 5′- TTTTTAGATATATAAATTTTTTTTTT-3′ (cg18752527) and fwd/seq: 5′-GTTATTTATAAAGGAGGGTGAGATTA-3′, rev: 5′-Bio-AACCACTACTCCTATAAAACCCCAC-3′ (cg16529483/cg24496423). A detailed list of PCR primers and programs is provided in Supplementary Table S4. Specificity of the PCR was verified by agarose gel electrophoresis including a negative control. Pyrosequencing was conducted on a PyroMark Q24 according to standard protocol using PyroMark Gold Reagents (both Qiagen). Each sample was measured in triplicates; an intra-sample deviation of ≥3% led to the exclusion of the deviating measurement. For each site, measurements of DNA with known methylation levels of 0%, 25%, 50%, 75% and 100% were obtained (Epitect Control DNA, Qiagen). Correlations between the 450 K dataset and pyrosequencing were tested using the Spearman’s correlation test.

### Pyrosequencing-based validation and replication in whole blood

DNA was prepared from EDTA tubes using QIAamp DNA Blood Maxi Kit (Qiagen) according to manufacturer’s protocol. Afterwards, bisulfite conversion and pyrosequencing was carried out as described above.

## Electronic supplementary material

Supplementary Information
