# Supplementary material for: DNA methylation signatures of chronic alcohol dependence in purified CD3+ T-cells of patients undergoing alcohol treatment
Source: Sci Rep. 2017 Jul 26;7:6605. doi: 10.1038/s41598-017-06847-z (PMC5529570; doi:10.1038/s41598-017-06847-z)
Supplement: Supplementary file 1 — Supplementary Information [file 41598_2017_6847_MOESM1_ESM.doc]

# DNA methylation signatures of chronic alcohol dependence in purified CD3^+^ T-cells of patients undergoing alcohol treatment

Christof Brückmann, Sumaiya A. Islam, Julia L. MacIsaac, Alexander M. Morin, Kathrin N. Karle, Adriana Di Santo, Richard Wüst, Immanuel Lang, Anil Batra, Michael S. Kobor, and Vanessa Nieratschker

# *Supplementary Information*

**Supplementary methods**

**DNA methylation array data quality control and normalization:** Quality control, processing and differential DNAm analysis of 450K array data was performed as previously described.^1,2^ Briefly, raw intensity values from the arrays were imported into Illumina GenomeStudio V2011.1 software and subjected to initial quality control checks for array staining, extension and bisulfite conversion followed by color correction and background adjustment using control probes contained on the 450K array. Subsequent processing and analysis were performed in R Version 3.2.1 (http://www.r-project.org). Profiles from 65 probes targeting single nucleotide polymorphisms (SNPs) were used to ensure T1 and T2 samples were indeed matched from the same individual. The 65 SNP probes were also filtered out of the dataset. Additional probe filtering was performed in which poor performing probes including those with detection *P*-values greater than 0.01, probes with missing beta values, and probes for which less than three beads contributed to the signal in any sample were eliminated (a total of 13 903). Recent re-annotation of the Illumina 450K array^3^ was used to filter 19 343 probes that are known to be polymorphic at the target CpG. Probes which have nonspecific in silico binding to the sex chromosomes were assessed in a post-hoc analysis following differential DNAm analysis to ensure they did not overlap with identified hits.^3^ Together, quality control checks eliminated 33 311 probes, leaving a total of 452 266 probes for further analysis. Following quality control processing, quantile normalization was conducted using the lumi R package^4^ after assessment using the quantro package indicated that quantile normalization was appropriate for this dataset.^5^ Differences between Type I and Type II probes on the 450K array were normalized using Subset-quantile Within Array Normalization (SWAN).^6^ ComBat^7^ was then used to remove chip and row effects, while protecting sample group. Removal of technical variation was assessed by principal component analysis (PCA).

**Blood cell type deconvolution:** To test for potential contamination of bead-purified CD3^+^ T-cell samples by other blood cell types, a well-established algorithm was used to bioinformatically estimate cell type composition based on underlying reference DNAm profiles.^8,9^ In addition, the 450K data was subjected to advanced DNA methylation age analysis in blood using a publicly available DNA methylation age predictor tool in order to obtain predicted abundance measures of additional blood cell types including plasma blasts, CD8^+^CD28^-^CD45RA^-^ (memory and effector) T-cells, naïve CD8^+^ T-cells and naïve CD4^+^ T-cells.^10^ Upon detection of potential non-T-cell contamination in a fraction of samples, we removed this cell-type composition variation by regressing probewise DNAm on estimated cell type proportions, as previously described.^11^ The residuals of each regression model were applied to the mean value of each data series to obtain the ‘corrected’ DNAm data. PCA was subsequently used to check that the presence of the cell type proportions in DNAm variation was minimal in the corrected dataset. PCA was additionally used to check for correlation of other known meta-variables (i.e. sample group, age, daily smoking) with the underlying DNAm patterns of the uncorrected and corrected 450K datasets, respectively. Note that for all PCA analyses, the top-ranking PC (denoted as PC0) was negated as it is not informative of inter-individual variance in the DNAm data.^12^

**Differential methylation analyses of 450K dataset:** The cell-type corrected 450K dataset was subsetted into controls versus patients (T1), patients (T1) versus patients (T2) and controls versus patients (T2) sample sets, respectively, prior to differential DNAm analysis. In the genome-wide analyses, differentially methylated probes were identified using the R limma package’s moderated t-statistics with empirical Bayesian variance estimation.^13^ Specifically, in the comparison of controls versus patients (T1), a linear model was fit for each probe’s DNAm measures with sample group as the main effect, adjusted for age and smoking levels. In the comparison of patients (T1) and patients (T2) samples, differentially methylated probes were identified using paired testing in linear regression analysis. For both of these comparisons, differentially methylated regions (DMRs) were detected using DMRcate package which uses the moderated t-statistics generated in their respective limma analyses.^14^ In the comparison of controls versus patients (T2), we sought to assess which differentially methylated sites between controls and patients (T1) exhibited reversion in the patient (T2) samples such that their DNAm levels were comparable to controls. To address this, we specifically tested the 59 hits identified between controls versus patients (T1) (FDR < 0.1 and DNAm difference > 5%) by fitting individual linear models for each of the 59 probes. For all tests, the resulting *P*-values were adjusted using the Benjamini-Hochberg False Discovery Rate (FDR) method.^15^ All statistical analyses were performed on transformed M-values.^16^

**Questionnaire evaluation:** The AUDIT score is the sum of all 10 items of the questionnaire. The GSI score represents the sum of all the subscales of the SCL-90-R divided by the number of answered items (usually 90). For the OCDS score, the higher value of four item pairs (Items 1 and 2, 7 and 8, 9 and 10, and 12 and 13) was added up with the remaining items, leading to a potential range of 0 to 40. Up to one missing item was allowed and replaced by adding the mean of all other items.


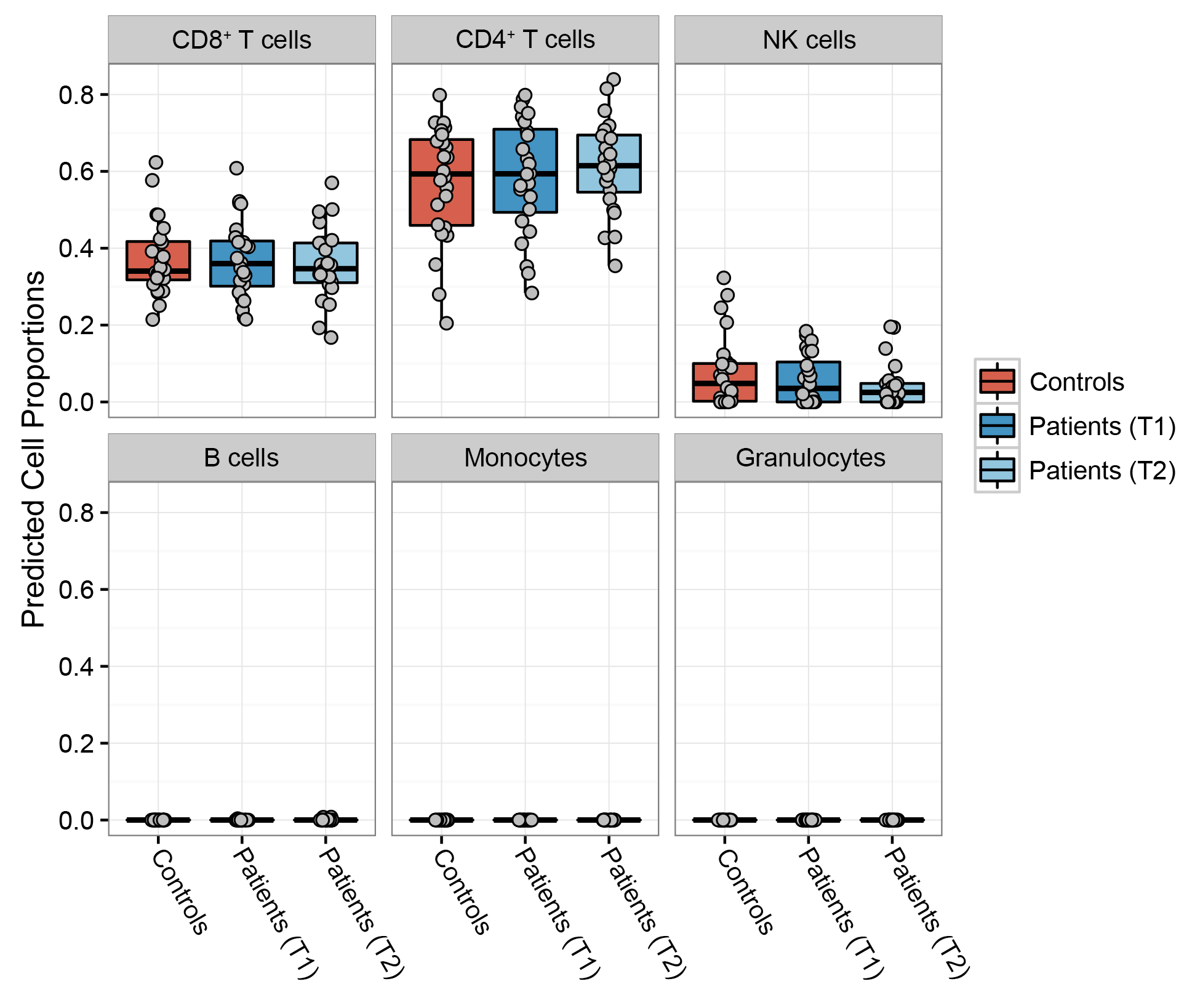


**Supplementary Figure S1.** **Estimations of blood cell proportions in samples based on underlying reference DNAm profiles.** Estimates were predicted using the Houseman blood cell deconvolution algorithm. There was no statistically significant association between predicted proportions of any cell type and sample group (Mann-Whitney U test for comparison of controls and patients (T1) or controls and patients (T2); Wilcoxon signed-rank test for comparison of matched patients (T1) and patients (T2) samples).


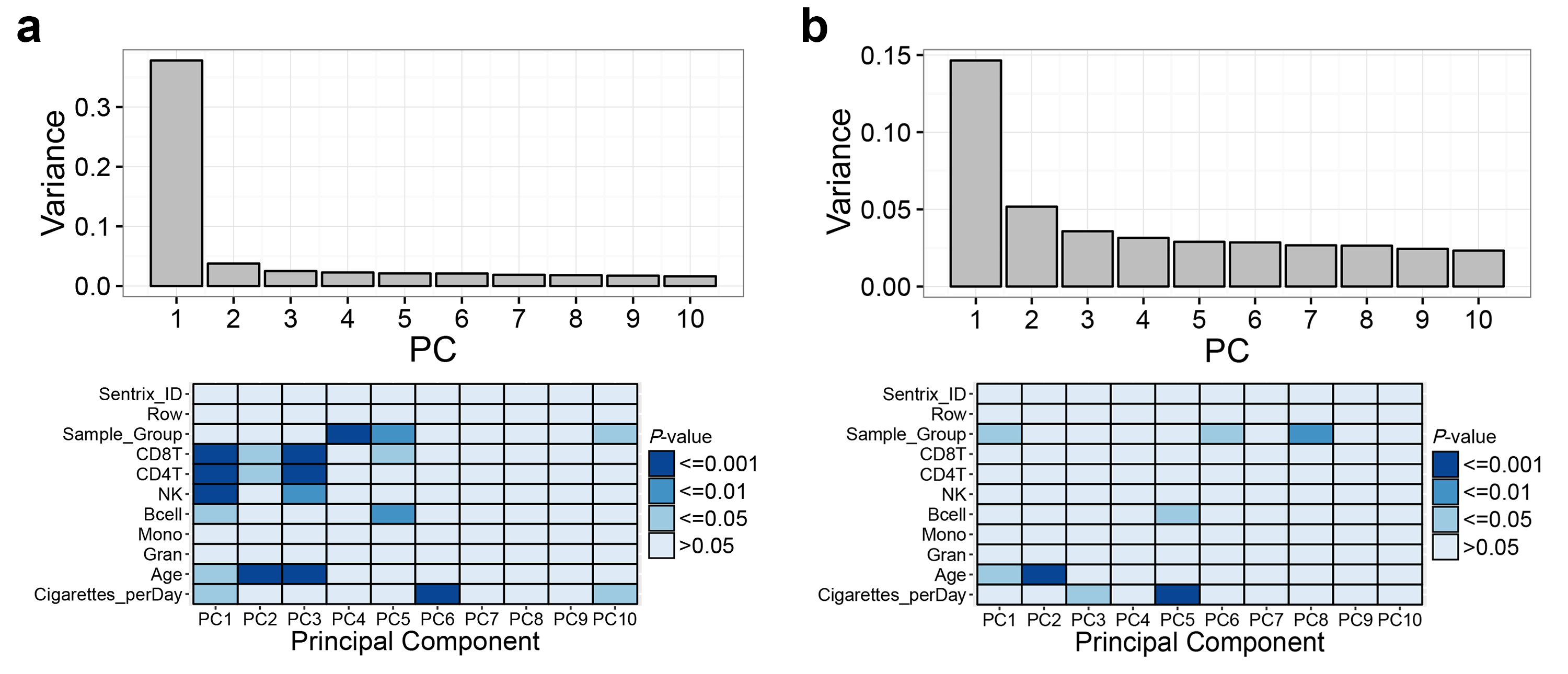


**Supplementary Figure S2.** **Principal component analyses before and after regression-based adjustment of the 450K data.** PCA showing the correlation of known phenotypic and technical variables to the top 10 principal components, each representing an incremental proportion of the variance in the methylation data. a) Top 10 PCs in unadjusted 450K dataset (representing 60% of the DNAm variance) and b) top 10 PCs in the adjusted 450K dataset (representing 45% of the DNAm variance).

| **Supplementary Table S1.** Differentially methylated sites between Controls and Patients (T1) | | | | | | | | |
| --- | --- | --- | --- | --- | --- | --- | --- | --- |
| # | Probe ID | Gene | Region | Average beta Controls | Average  beta  Patients (T1) | Δ-beta | *P*-Value | BH-adjusted  *P*-Value |
| 1 | cg18752527 | HECW2 | intragenic | 0.342 | 0.276 | 0.066 | 4.30E-07 | 0.0213 |
| 2 | cg08109624 | None | intergenic | 0.760 | 0.817 | -0.057 | 8.15E-07 | 0.0234 |
| 3 | cg10168086 | None | intergenic | 0.535 | 0.484 | 0.051 | 1.24E-06 | 0.0256 |
| 4 | cg07280807 | None | intergenic | 0.755 | 0.822 | -0.068 | 2.44E-06 | 0.0366 |
| 5 | cg12173150 | None | intergenic | 0.321 | 0.385 | -0.064 | 3.02E-06 | 0.0370 |
| 6 | cg01059398 | TNFSF10 | intragenic | 0.261 | 0.209 | 0.052 | 1.07E-05 | 0.0627 |
| 7 | cg17940902 | HLA-DMA | promoter | 0.399 | 0.450 | -0.051 | 1.19E-05 | 0.0640 |
| 8 | cg22778903 | MX2 | intragenic | 0.304 | 0.355 | -0.051 | 1.34E-05 | 0.0666 |
| 9 | cg14612335 | SKIL | promoter | 0.423 | 0.368 | 0.055 | 1.38E-05 | 0.0666 |
| 10 | cg11580026 | None | intergenic | 0.600 | 0.549 | 0.051 | 1.51E-05 | 0.0691 |
| 11 | cg12284098 | MYOM2 | intragenic | 0.534 | 0.477 | 0.056 | 1.54E-05 | 0.0691 |
| 12 | cg26091609 | CTLA4 | intragenic | 0.578 | 0.518 | 0.060 | 1.59E-05 | 0.0691 |
| 13 | cg09768654 | SRPK3 | promoter | 0.374 | 0.466 | -0.092 | 1.65E-05 | 0.0691 |
| 14 | cg06851207 | PNMAL1 | promoter | 0.528 | 0.617 | -0.089 | 1.84E-05 | 0.0691 |
| 15 | cg14702960 | None | intergenic | 0.742 | 0.689 | 0.052 | 1.92E-05 | 0.0691 |
| 16 | cg00449728 | MAPRE2 | intragenic | 0.750 | 0.693 | 0.057 | 2.98E-05 | 0.0702 |
| 17 | cg22851561 | ELMSAN1 | intragenic | 0.432 | 0.380 | 0.052 | 3.00E-05 | 0.0702 |
| 18 | cg02536838 | ANGPT1 | promoter | 0.605 | 0.530 | 0.075 | 3.14E-05 | 0.0702 |
| 19 | cg15841511 | None | intergenic | 0.729 | 0.788 | -0.059 | 3.42E-05 | 0.0706 |
| 20 | cg24392939 | CRYBG3 | intragenic | 0.562 | 0.510 | 0.052 | 3.62E-05 | 0.0725 |
| 21 | cg12761472 | CEP85L | promoter | 0.621 | 0.566 | 0.055 | 4.13E-05 | 0.0754 |
| 22 | cg02652579 | SYNGAP1 | promoter | 0.623 | 0.563 | 0.059 | 4.17E-05 | 0.0758 |
| 23 | cg22865905 | SNORA69 | three_plus | 0.794 | 0.743 | 0.051 | 4.26E-05 | 0.0764 |
| 24 | cg27201673 | PNMAL1 | promoter | 0.213 | 0.263 | -0.050 | 5.41E-05 | 0.0778 |
| 25 | cg04936619 | C17orf75 | intragenic | 0.314 | 0.245 | 0.069 | 5.88E-05 | 0.0778 |
| 26 | cg11121969 | PCBP3 | promoter | 0.691 | 0.627 | 0.064 | 6.26E-05 | 0.0778 |
| 27 | cg00246693 | ARHGAP42 | Promoter | 0.340 | 0.393 | -0.053 | 7.10E-05 | 0.0778 |
| # | Probe ID | Gene | Region | Average beta Controls | Average  beta  Patients (T1) | Δ-beta | *P*-Value | BH-adjusted  *P*-Value |
| 28 | cg10399005 | None | intergenic | 0.776 | 0.833 | -0.057 | 7.11E-05 | 0.0778 |
| 29 | cg16529483 | SRPK3 | promoter | 0.252 | 0.357 | -0.105 | 7.18E-05 | 0.0780 |
| 30 | cg01220513 | SH3KBP1 | intragenic | 0.506 | 0.454 | 0.051 | 8.08E-05 | 0.0791 |
| 31 | cg26926002 | None | intergenic | 0.719 | 0.777 | -0.058 | 8.10E-05 | 0.0791 |
| 32 | cg14544087 | MIR155HG | intragenic | 0.290 | 0.227 | 0.063 | 8.64E-05 | 0.0791 |
| 33 | cg20893919 | TRPC3 | intragenic | 0.703 | 0.754 | -0.051 | 9.23E-05 | 0.0801 |
| 34 | cg18682028 | FYCO1 | intragenic | 0.394 | 0.338 | 0.056 | 9.24E-05 | 0.0801 |
| 35 | cg04362790 | None | intergenic | 0.697 | 0.644 | 0.052 | 9.32E-05 | 0.0801 |
| 36 | cg09060654 | LIPA | intragenic | 0.578 | 0.656 | -0.079 | 9.51E-05 | 0.0801 |
| 37 | cg02451774 | NBPF8 | intragenic | 0.431 | 0.483 | -0.053 | 9.98E-05 | 0.0806 |
| 38 | cg18723276 | USP29 | promoter | 0.723 | 0.774 | -0.051 | 0.0001 | 0.0819 |
| 39 | cg13180722 | None | intergenic | 0.338 | 0.401 | -0.062 | 0.0001 | 0.0830 |
| 40 | cg12230162 | SRPK3 | promoter | 0.357 | 0.463 | -0.105 | 0.0001 | 0.0835 |
| 41 | cg18890544 | None | intergenic | 0.846 | 0.905 | -0.059 | 0.0001 | 0.0839 |
| 42 | cg24496423 | SRPK3 | promoter | 0.309 | 0.393 | -0.084 | 0.0001 | 0.0854 |
| 43 | cg02661764 | None | intergenic | 0.419 | 0.360 | 0.059 | 0.0001 | 0.0867 |
| 44 | cg01400671 | None | intergenic | 0.409 | 0.345 | 0.064 | 0.0001 | 0.0874 |
| 45 | cg13609457 | None | intergenic | 0.577 | 0.521 | 0.056 | 0.0002 | 0.0897 |
| 46 | cg25880958 | None | intergenic | 0.591 | 0.645 | -0.054 | 0.0002 | 0.0898 |
| 47 | cg18376497 | INPP4B | intragenic | 0.286 | 0.223 | 0.064 | 0.0002 | 0.0919 |
| 48 | cg13784312 | RAPGEF1 | intragenic | 0.187 | 0.136 | 0.051 | 0.0002 | 0.0928 |
| 49 | cg07135405 | MIR1914 | three_plus | 0.540 | 0.394 | 0.146 | 0.0002 | 0.0928 |
| 50 | cg20475486 | None | intergenic | 0.702 | 0.759 | -0.058 | 0.0002 | 0.0936 |
| 51 | cg11858450 | CCDC105 | intragenic | 0.709 | 0.762 | -0.053 | 0.0002 | 0.0940 |
| 52 | cg05927817 | None | intergenic | 0.726 | 0.787 | -0.061 | 0.0002 | 0.0940 |
| 53 | cg00306893 | None | intergenic | 0.737 | 0.675 | 0.062 | 0.0002 | 0.0940 |
| 54 | cg10365886 | TNXB | intragenic | 0.566 | 0.672 | -0.105 | 0.0002 | 0.0947 |
| 55 | cg27503950 | None | intergenic | 0.633 | 0.696 | -0.063 | 0.0002 | 0.0952 |
| # | Probe ID | Gene | Region | Average beta Controls | Average  beta  Patients (T1) | Δ-beta | *P*-Value | BH-adjusted  *P*-Value |
| 56 | cg01089001 | GALNT18 | intragenic | 0.317 | 0.382 | -0.065 | 0.0002 | 0.0953 |
| 57 | cg12564698 | GAL | three_plus | 0.312 | 0.261 | 0.051 | 0.0002 | 0.0953 |
| 58 | cg16197188 | NRG3 | intragenic | 0.723 | 0.672 | 0.051 | 0.0003 | 0.0995 |
| 59 | cg04088338 | None | intergenic | 0.430 | 0.378 | 0.052 | 0.0003 | 0.0999 |
| Abbreviations: Average beta, mean methylation values (%); Benjamini-Hochberg (BH) adjusted P-value. | | | | | | | | |

| **Supplementary Table S2.** Top listed hits detected by both site-specific and DMRcate analysis. | | | | | | | | |
| --- | --- | --- | --- | --- | --- | --- | --- | --- |
| Probe ID | Gene | DMR | Position | Average beta Controls | Average beta Patients (T1) | Δ-beta | *P-*Value | FDR |
| cg16529483 | *SRPK3* | chrX:153046175-153047707 | 153046451 | 0.252 | 0.357 | -0.105 | 3.52E-23 | 5.90E-19 |
| cg24496423 | *SRPK3* | chrX:153046175-153047707 | 153046480 | 0.309 | 0.393 | -0.084 | 2.84E-23 | 4.94E-19 |
| cg12230162 | *SRPK3* | chrX:153046175-153047707 | 153046482 | 0.357 | 0.463 | -0.105 | 2.80E-23 | 4.94E-19 |
| cg09768654 | *SRPK3* | chrX:153046175-153047707 | 153046386 | 0.374 | 0.466 | -0.092 | 6.72E-23 | 1.01E-18 |
| cg18890544 |  | chr1:242220301-242220925 | 242220538 | 0.846 | 0.905 | -0.059 | 1.75E-18 | 8.88E-15 |
| cg08109624 |  | chr1:242220301-242220925 | 242220925 | 0.760 | 0.817 | -0.057 | 1.69E-19 | 1.02E-15 |
| cg27503950 |  | chr6:160023581-160024144 | 160024002 | 0.633 | 0.696 | -0.063 | 2.92E-15 | 6.57E-12 |
| cg09060654 | *LIPA* | chr10:90985055-90985062 | 90985062 | 0.578 | 0.656 | -0.079 | 1.96E-07 | 4.53E-05 |
| Abbreviations: Average beta, mean methylation values (%); FDR, Benjamini-Hochberg False Discovery Rate; DMR, differentially methylated region. | | | | | | | | |

| **Supplementary Table S3.** Differentially methylated sites between Patients (T1) and Patients (T2) | | | | | | | | |
| --- | --- | --- | --- | --- | --- | --- | --- | --- |
| # | Probe ID | Gene | Region | Average  beta  Patients (T1) | Average  beta  Patients (T2) | Δ-beta | *P*-Value | BH-adjusted  *P*-Value |
| 1 | cg15500907 | LAMA4 | intragenic | 0.485 | 0.542 | -0.056 | 1.01E-06 | 0.0323 |
| 2 | cg05266321 | CCR2 | intragenic | 0.545 | 0.606 | -0.061 | 4.63E-06 | 0.0487 |
| 3 | cg13279700 | C6orf10 | intragenic | 0.481 | 0.544 | -0.063 | 1.76E-05 | 0.0561 |
| 4 | cg14054990 | KRTAP19-5 | promoter | 0.431 | 0.482 | -0.052 | 1.84E-05 | 0.0565 |
| 5 | cg21049302 | None | intergenic | 0.466 | 0.522 | -0.056 | 1.98E-05 | 0.0565 |
| 6 | cg17022548 | NRG2 | intragenic | 0.204 | 0.258 | -0.054 | 1.99E-05 | 0.0565 |
| 7 | cg22472360 | TRIO | intragenic | 0.514 | 0.569 | -0.055 | 2.09E-05 | 0.0569 |
| 8 | cg07920414 | RIMS3 | intragenic | 0.438 | 0.493 | -0.055 | 2.18E-05 | 0.0572 |
| 9 | cg04088338 | None | intergenic | 0.378 | 0.429 | -0.051 | 2.54E-05 | 0.0590 |
| 10 | cg12240358 | HOMER2 | intragenic | 0.462 | 0.519 | -0.057 | 2.68E-05 | 0.0590 |
| 11 | cg09712306 | AURKA | intragenic | 0.602 | 0.660 | -0.058 | 3.48E-05 | 0.0605 |
| 12 | cg07939743 | None | intergenic | 0.289 | 0.341 | -0.052 | 3.50E-05 | 0.0605 |
| 13 | cg00803692 | CCR5 | promoter | 0.370 | 0.424 | -0.054 | 3.73E-05 | 0.0620 |
| 14 | cg10177030 | SNORD12 | three_plus | 0.419 | 0.472 | -0.053 | 3.85E-05 | 0.0627 |
| 15 | cg15439110 | None | intergenic | 0.444 | 0.525 | -0.080 | 3.93E-05 | 0.0628 |
| 16 | cg20385229 | SLIRP | intragenic | 0.392 | 0.444 | -0.052 | 4.13E-05 | 0.0628 |
| 17 | cg02393640 | LUZP6 | intragenic | 0.390 | 0.443 | -0.052 | 5.63E-05 | 0.0668 |
| 18 | cg17863551 | CD177 | promoter | 0.419 | 0.478 | -0.059 | 6.27E-05 | 0.0670 |
| 19 | cg15279541 | None | intergenic | 0.388 | 0.439 | -0.051 | 7.14E-05 | 0.0677 |
| 20 | cg20171999 | RRS1 | three_plus | 0.403 | 0.474 | -0.070 | 8.93E-05 | 0.0680 |
| 21 | cg20559385 | None | intergenic | 0.428 | 0.479 | -0.052 | 9.43E-05 | 0.0680 |
| 22 | cg21429780 | MAML3 | intragenic | 0.493 | 0.545 | -0.052 | 0.0001 | 0.0680 |
| 23 | cg01482790 | HNRNPM | intragenic | 0.289 | 0.339 | -0.050 | 0.0001 | 0.0681 |
| 24 | cg20684197 | FGF1 | intragenic | 0.395 | 0.445 | -0.051 | 0.0001 | 0.0684 |
| 25 | cg04279139 | MANSC4 | promoter | 0.410 | 0.461 | -0.051 | 0.0001 | 0.0688 |
| 26 | cg16853860 | PSMB9 | intragenic | 0.272 | 0.332 | -0.060 | 0.0001 | 0.0696 |
| 27 | cg27062514 | CTR9 | intragenic | 0.463 | 0.526 | -0.064 | 0.0001 | 0.0721 |
| # | Probe ID | Gene | Region | Average  beta  Patients (T1) | Average  beta  Patients (T2) | Δ-beta | *P*-Value | BH-adjusted *P*-Value |
| 28 | cg09931909 | MB21D1 | intragenic | 0.420 | 0.497 | -0.077 | 0.0001 | 0.0735 |
| 29 | cg13340231 | ZNF704 | intragenic | 0.528 | 0.583 | -0.055 | 0.0002 | 0.0751 |
| 30 | cg10035831 | RPTOR | intragenic | 0.446 | 0.503 | -0.057 | 0.0002 | 0.0753 |
| 31 | cg13927756 | MYO10 | intragenic | 0.468 | 0.524 | -0.056 | 0.0002 | 0.0754 |
| 32 | cg08749576 | None | intergenic | 0.627 | 0.684 | -0.058 | 0.0002 | 0.0761 |
| 33 | cg15484808 | RPS18 | intragenic | 0.480 | 0.534 | -0.054 | 0.0002 | 0.0811 |
| 34 | cg12802876 | None | intergenic | 0.359 | 0.418 | -0.059 | 0.0002 | 0.0828 |
| 35 | cg03548415 | None | intergenic | 0.422 | 0.473 | -0.051 | 0.0003 | 0.0853 |
| 36 | cg20547015 | PPP1CC | intragenic | 0.453 | 0.517 | -0.064 | 0.0003 | 0.0862 |
| 37 | cg23214895 | None | intergenic | 0.569 | 0.620 | -0.051 | 0.0003 | 0.0878 |
| 38 | cg12478092 | CCDC116 | promoter | 0.510 | 0.573 | -0.063 | 0.0003 | 0.0879 |
| 39 | cg15683542 | MIPEP | intragenic | 0.694 | 0.747 | -0.053 | 0.0003 | 0.0883 |
| 40 | cg09514545 | MIR525 | three_plus | 0.442 | 0.501 | -0.060 | 0.0004 | 0.0908 |
| 41 | cg01789743 | NID1 | intragenic | 0.499 | 0.552 | -0.053 | 0.0004 | 0.0910 |
| 42 | cg18524114 | None | intergenic | 0.339 | 0.389 | -0.050 | 0.0005 | 0.0933 |
| 43 | cg04410448 | ZC2HC1B | intragenic | 0.491 | 0.541 | -0.051 | 0.0005 | 0.0949 |
| 44 | cg13714407 | RAPGEF1 | intragenic | 0.367 | 0.426 | -0.059 | 0.0005 | 0.0953 |
| 45 | cg27367066 | None | intergenic | 0.455 | 0.510 | -0.054 | 0.0006 | 0.0967 |
| 46 | cg26837708 | YBX1 | intragenic | 0.388 | 0.445 | -0.058 | 0.0006 | 0.0967 |
| 47 | cg14817867 | PRPSAP2 | intragenic | 0.419 | 0.471 | -0.052 | 0.0006 | 0.0971 |
| 48 | cg13598358 | PPP1CC | intragenic | 0.362 | 0.418 | -0.056 | 0.0006 | 0.0978 |
| Abbreviations: Average beta, mean methylation values (%); Benjamini-Hochberg (BH) adjusted P-value. | | | | | | | | |


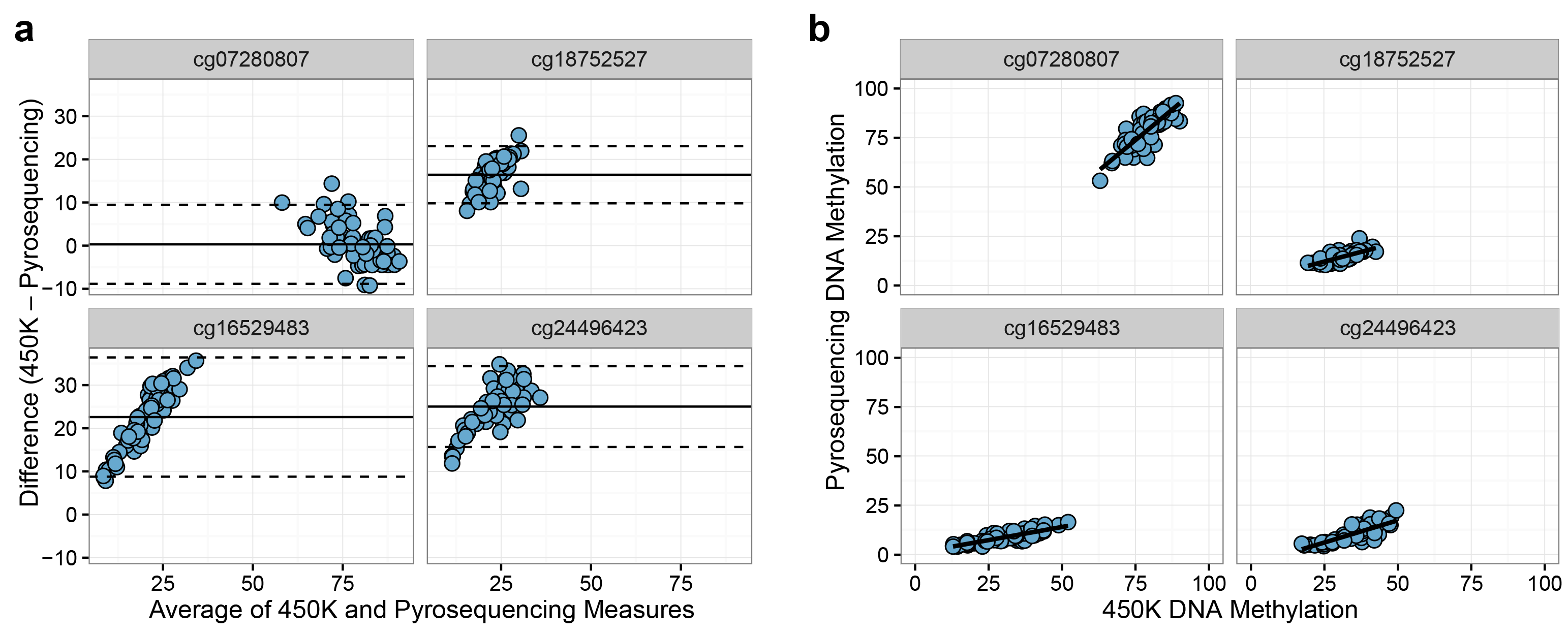


**Supplementary Figure S3.** **Correlations between 450K array and pyrosequencing measures.** a) Bland-Altman plots for verified CpGs show a slightly biased agreement between 450K dataset and pyrosequencing measures. b) Strong positive correlation between 450K and pyrosequencing measures for cg07280807 (Spearman r_s_ = 0.85,
*P* = 2E-16), cg18752527 (r_s_ = 0.71, *P* = 3E-12), cg16529483 (r_s_ = 0.79, *P* = 4E-16) and cg24496423 (r_s_ = 0.80, *P* = 2E-16).


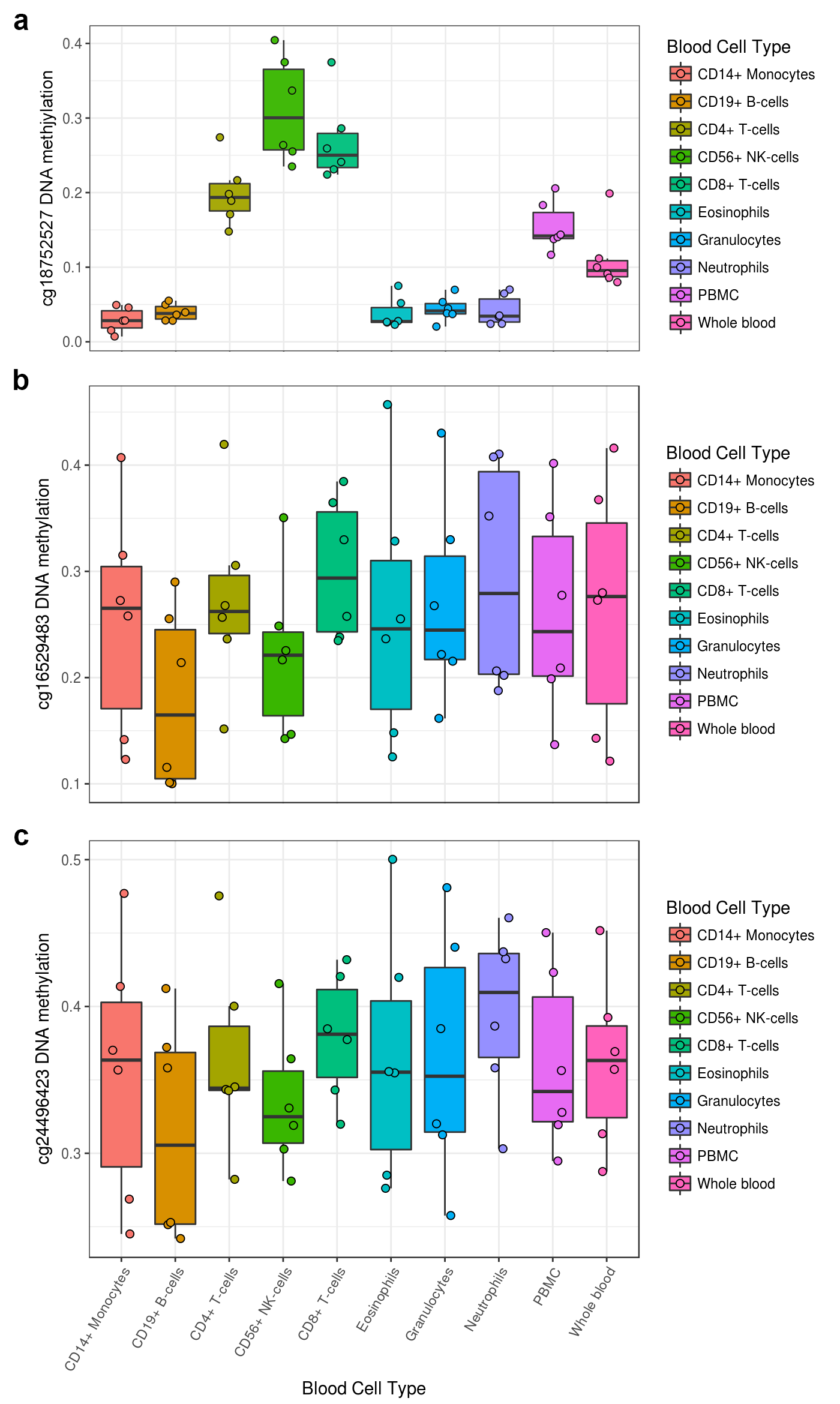


**Supplementary Figure S4. Blood cell type associations of 3 examined CpG sites.** a) DNA methylation of cg18752527 in the HECW2 gene was significantly associated with CD4^+^ and CD8^+^ T cells, along with NK cells, as determined by differential DNAm testing using a previous 450K dataset of purified blood cell types^17^ (P = 7.6E-15, ANOVA). DNA methylation of cg16529483 (b) and cg24496423 (c) in the SRPK3 gene were not significantly associated with any cell type (P > 0.6, ANOVA).

| **Supplementary Table S4.** Primers and PCR programs for validation and replication. | |
| --- | --- |
| **cg07280807 (intergenic)**  fwd: 5’-GTTATGGTTGGGTTTTTGGG-3’  rev: 5’-Bio-CCTATCTCCTCAAACAAAAACTAAAAA-3’  seq: 5’-AGTTAGGGATTATAGTGTAGTTG-3’  Amplicon length: 156 bp  coordinates: chr14:70,317,178-70,317,333  Note: The amplicon contains 3 CpG sites, of which the third is cg07280807 | **PCR program:**  95°C – 15 min  -------------------  45 cycles:  94°C – 30 sec  50°C – 30 sec  72°C – 30 sec  -------------------  72°C – 10 min  4°C – hold |
| **cg18752527 (*HECW2*)**  fwd: 5’-GTGTTTGTGGGAATGTTTTTTATA-3’  rev: 5’-Bio- CACACTACACTTTCATTTTCTATCAA-3’  seq: 5’- TTTTTAGATATATAAATTTTTTTTTT-3’  Amplicon length: 135 bp  coordinates: chr2:197,132,798-197,132,932 | **PCR program:**  95°C – 15 min  -------------------  45 cycles:  94°C – 30 sec  50°C – 30 sec  72°C – 30 sec  -------------------  72°C – 10 min  4°C – hold |
| **cg16529483 / cg24496423 (*SRPK3*)**  fwd/seq: 5’-GTTATTTATAAAGGAGGGTGAGATTA-3’  rev: 5’-Bio-AACCACTACTCCTATAAAACCCCAC-3’  Amplicon length: 85 bp  coordinates: chrX:153,046,424-153,046,508  Note: The amplicon contains 5 CpG sites, of which the first is cg16529483 and the fourth is cg24496423. Due to CpG sites in the primer binding area, the primers contain 1 (fwd) and 2 (rev) mismatches, which are highlighted underlined. | **PCR program:**  95°C – 15 min  -------------------  45 cycles:  94°C – 30 sec  48°C – 30 sec  72°C – 30 sec  -------------------  72°C – 10 min  4°C – hold |
| Abbreviations: fwd, forward primer; rev, reverse primer; seq, sequencing primer; Bio, biotin-modification; bp, basepair. | |

# Supplementary References

1 Esposito, E. A. *et al.* Differential DNA methylation in peripheral blood mononuclear cells in adolescents exposed to significant early but not later childhood adversity. *Dev Psychopathol* **28**, 1385-1399, doi:10.1017/S0954579416000055 (2016).

2 De Souza, R. A. *et al.* DNA methylation profiling in human Huntington's disease brain. *Human molecular genetics* **25**, 2013-2030, doi:10.1093/hmg/ddw076 (2016).

3 Price, M. E. *et al.* Additional annotation enhances potential for biologically-relevant analysis of the Illumina Infinium HumanMethylation450 BeadChip array. *Epigenetics Chromatin* **6**, 4, doi:10.1186/1756-8935-6-4 (2013).

4 Du, P., Kibbe, W. A. & Lin, S. M. lumi: a pipeline for processing Illumina microarray. *Bioinformatics* **24**, 1547-1548, doi:10.1093/bioinformatics/btn224 (2008).

5 Hicks, S. C. & Irizarry, R. A. quantro: a data-driven approach to guide the choice of an appropriate normalization method. *Genome Biol* **16**, 117, doi:10.1186/s13059-015-0679-0 (2015).

6 Maksimovic, J., Gordon, L. & Oshlack, A. SWAN: Subset-quantile within array normalization for illumina infinium HumanMethylation450 BeadChips. *Genome Biol* **13**, R44, doi:10.1186/gb-2012-13-6-r44 (2012).

7 Johnson, W. E., Li, C. & Rabinovic, A. Adjusting batch effects in microarray expression data using empirical Bayes methods. *Biostatistics* **8**, 118-127, doi:10.1093/biostatistics/kxj037 (2007).

8 Houseman, E. A. *et al.* DNA methylation arrays as surrogate measures of cell mixture distribution. *BMC Bioinformatics* **13**, 86, doi:10.1186/1471-2105-13-86 (2012).

9 Koestler, D. C. *et al.* Blood-based profiles of DNA methylation predict the underlying distribution of cell types: a validation analysis. *Epigenetics* **8**, 816-826, doi:10.4161/epi.25430 (2013).

10 Horvath, S. DNA methylation age of human tissues and cell types. *Genome Biol* **14**, R115, doi:10.1186/gb-2013-14-10-r115 (2013).

11 Jones, M. J., Islam, S. A., Edgar, R. D. & Kobor, M. S. Adjusting for Cell Type Composition in DNA Methylation Data Using a Regression-Based Approach. *Methods in molecular biology*, doi:10.1007/7651_2015_262 (2015).

12 Lam, L. L. *et al.* Factors underlying variable DNA methylation in a human community cohort. *Proceedings of the National Academy of Sciences of the United States of America* **109 Suppl 2**, 17253-17260, doi:10.1073/pnas.1121249109 (2012).

13 Smyth, G. K. Linear models and empirical bayes methods for assessing differential expression in microarray experiments. *Stat Appl Genet Mol Biol* **3**, Article3, doi:10.2202/1544-6115.1027 (2004).

14 Peters, T. J. *et al.* De novo identification of differentially methylated regions in the human genome. *Epigenetics Chromatin* **8**, 6, doi:10.1186/1756-8935-8-6 (2015).

15 Benjamini, Y. & Hochberg, Y. Controlling the false discovery rate: a practical and powerful approach to multiple testing. *Journal of the royal statistical society. Series B (Methodological)*, 289-300 (1995).

16 Du, P. *et al.* Comparison of Beta-value and M-value methods for quantifying methylation levels by microarray analysis. *BMC Bioinformatics* **11**, 587, doi:10.1186/1471-2105-11-587 (2010).

17 Reinius, L. E. *et al.* Differential DNA methylation in purified human blood cells: implications for cell lineage and studies on disease susceptibility. *PloS one* **7**, e41361, doi:10.1371/journal.pone.0041361 (2012).
